# Tri-Co Robots for the future: an interview with Guang-Zhong Yang

**DOI:** 10.1093/nsr/nwac263

**Published:** 2022-11-25

**Authors:** Tiefeng Li

**Affiliations:** Tiefeng Li is a Professor at Zhejiang University

## Abstract

Future robots will effectively interact with the environments, tasks and other agents, either people or robots. Robots with coexisting designs can safely operate with human and environments. Cooperation enables robots to collaborate with other agents. Cognition enables the robots to perceive and predict the behaviour. The concept of Tri-Co (Coexisting–Cooperative–Cognitive) benefits future studies and applications of robots.

Professor Guang-Zhong Yang (FREng, FIEEE, FIET, FAIMBE) is Dean of the Institute of Medical Robotics, Shanghai Jiao Tong University (SJTU); he was also the co-founder and director of the Hamlyn Centre for Robotic Surgery, Imperial College London, as well as the founding editor of *Science Robotics*. Currently he is appointed full-time as chair professor of SJTU. Professor Yang's main research interests are in medical imaging, sensing and robotics. Prof. Yang discussed with *NSR* the interesting fields of Tri-Co Robots, as well as sharing ideas on his own research.

## ROBOTS IN THE RESEARCH FRONTIER AND PRACTICAL APPLICATION

***NSR:*** What role can robots play in scientific research and practical applications? Can you give some examples?

***Yang:*** The evolution of robotics has undergone decades of development, from early inspirational and curiosity-driven concepts in science-fiction movies to now ubiquitous deployment of robots in almost all aspects of our lives—from manufacturing, transport, logistics, agriculture, to surgery, synthetic biology and outer-space and deep-sea exploration. How to make robots that are as smart as humans, yet with super-human power, accuracy, dexterity and the ability to work in dirty, dull, dangerous or extreme environments has always been the dream of roboticists, as well as the general public.

Not only in engineering applications, robots also play an increasingly important role in scientific research. When I first launched *Science Robotics* in 2016 with Marcia McNutt, the former Editor-in-Chief of *Science*, we coined the motto of the journal as ‘Science for Robotics and Robotics for Science’. The first part of the sentence indicates how to take advantage of basic science advances and the latest technological breakthroughs to drive innovation and new development of robotics, whereas the second part of the motto is to anticipate how robots will transform the future development of basic sciences. We have already seen how robots are used to build complex deep-sea installations and support outer-space exploration, unravelling the complex structure of biological worlds from cellular to molecular levels. Future development of robotics will need to leverage significant advances from a range of basic science disciplines including materials science, biology, chemistry, physics, energy and medicine, as well as the more traditionally involved engineering topics such as computing, electronics and mechanical engineering.

**Figure 1:**
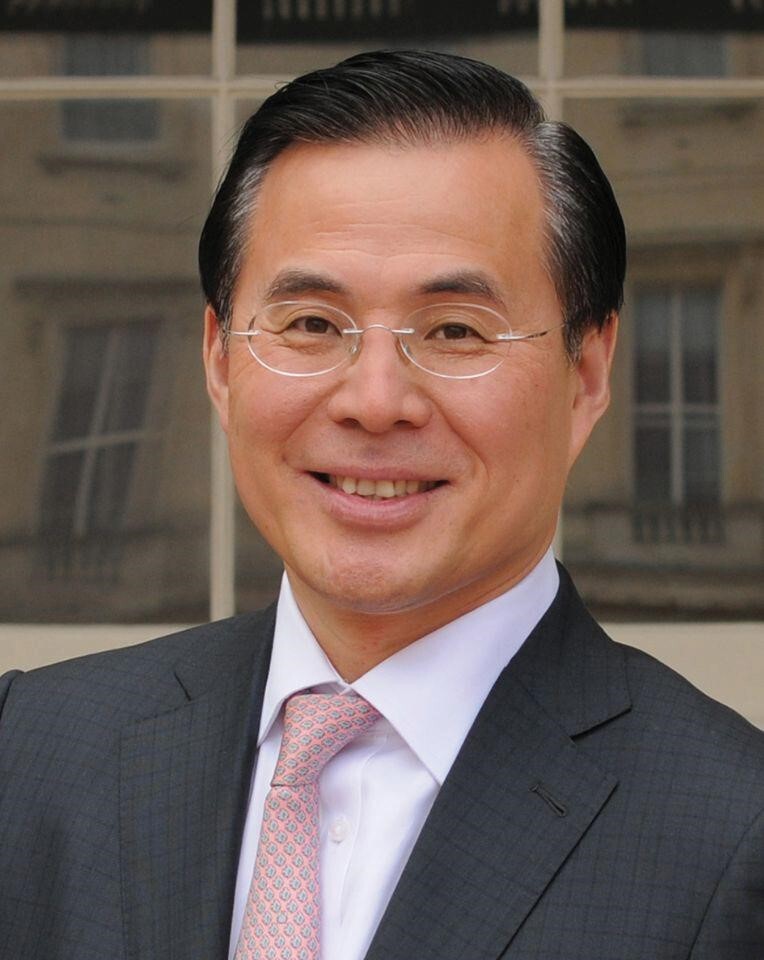
Professor Guang-Zhong Yang at Shanghai Jiao Tong University (*Image credit: Professor Guang-Zhong Yang*).

In China, there are many examples of how robots have helped underpin major scientific ventures including their use for our new space station and reaching the maximal depth of the Mariana Trench. In everyday life, robots are also increasingly used in hospitals; they are now common for minimally invasive surgery, rehabilitation, care for the elderly, as well as managing hospital logistics.

In China, there are many examples of how robots have helped underpin major scientific ventures including their use for our new space station and reaching the maximal depth of the Mariana Trench. In everyday life, robots are also increasingly used in hospitals; they are now common for minimally invasive surgery, rehabilitation, care for the elderly, as well as managing hospital logistics.

—Guang-Zhong Yang

The COVID-19 pandemic has also put a spotlight on robotics. We have already seen robots deployed for disinfection, delivering medications, food, measuring vital signs and assisting border controls. Prolonged social isolation and constant interruption of laboratory-based research work suggest that it is also time to consider seriously how robots should be used for laboratory automation, remote working and research collaboration.

***NSR:*** What are the frontiers in the international robotics research and application in recent years?

***Yang:*** Back in 2018, we published a review article in *Science Robotics* on the top 10 grand challenges of science robotics. These perhaps represent the general consensus of the community in terms of the new frontiers of robotics research and applications. Of course, our concerns are not limited to these 10 challenges; there are many other areas that are worth exploring.

For the 10 grand challenges we outlined, the first is about new materials and fabrication schemes. Why is this so important to the future of robotics? Well, when we talk about robots, what spring to mind are often gears, joints and electromechanical actuators. Indeed, these are fundamental to the precise motion control of many machines and robots that are in use today. The traditional concept of motor-driven robots based on mechanical transmission is the hallmark of most current robot designs. Many of the mechanical principles involved, however, are actually derived from the foundation established during the first Industrial Revolution. When combined with the latest micro-electronics, computer hardware and sensing technologies, we have created the contemporary robots. As we drive down the scale of robots, to sub-millimetre or sub-microns, the traditional route of using motors and gears may no longer be viable. We are exploring new materials such as artificial muscles, compliant materials for soft robots, and new manufacturing and fabrication schemes with the aim of creating multifunctional, power-efficient, compliant and autonomous robots that can emulate or compete with biological counterparts. New actuators made by these new materials can react to their environment by changing either material properties or shape, and react to temperature, chemical, optical, magnetic, electrical stimuli or mechanical loadings. With the development of smart materials and new fabrication schemes, it is also possible to have integrated sensing, actuation, control and even computation, thus establishing a completely new paradigm for the future of robotics.

The second challenge is in biohybrid and bio-inspired robotics. This is to translate fundamental biological principles into engineering design rules or integrate living components into synthetic structures to create robots that perform like natural systems. In fact, nature can provide us with much inspiration and many insights for building better robotic systems. In our article about the 10 grand challenges of robotics, we used a quote by Vogel: ‘As human technologies take on more of the characteristics of nature, nature becomes a more useful teacher.’ This is true in that we still have a lot to learn from birds about how to fly freely and elegantly, and understand how small fruit flies control and navigate so sensitively in their environment with only 100 000 neurons (compared to >80 billion in humans) and a limited vision system.

Other challenges include new power and energy—this involves not only new battery technologies, but also new power sources and energy-harvesting schemes for long-lasting operation of mobile robots; robot swarms that allow simpler, less expensive modular units to be reconfigured into a team to work together for more complex tasks; navigation and exploration in extreme environments that are not only unmapped but also poorly understood; effective use of AI for robots to learn, adapt and reason with common sense; and brain–computer interfacing for controlling neuroprostheses, functional electric stimulation devices and exoskeletons.

Among the 10 challenges, we also included social interaction and medical robotics as they are starting to have a greater impact on our daily lives and general wellbeing. Finally, we also emphasized the importance of ethics and security for responsible innovation in robotics. As we see increasing use of robots in our society, we must consider this very carefully and not too late. I believe that with increasing levels of autonomy and human–robot interaction, we must address potential regulatory, ethical and legal barriers, and the context of how robots are deployed in different aspects of our living environment.

***NSR:*** What issues in the field of robotics are you currently focusing on? What are the future directions of their development?

***Yang:*** My main work is in the field of medical robots and the Medical Robotics Institute that I established at Shanghai Jiao Tong University mainly focuses on surgical, assistive and hospital automation robots.

In the field of surgical robotics, since the initial research and development in the 1980s, it has undergone four different generations. The first generation of surgical robots is based on adapting industrial robotic arms for performing a surgical task such as bone milling in orthopaedic surgery. This is more about demonstrating the feasibility of using robots for surgery without considering some of the specific issues and subtleties encountered in clinical settings. The second-generation surgical robots, such as the Zeus and da Vinci robots, were designed from the ground up, addressing some of the key issues involved

Human–robot symbiosis has always been an important topic in robotics. As you know, it is a difficult task for robots to completely replace people in many fields, especially in complex environments. Hence, how to optimize the collective capability or behaviour is what Tri-Co Robots should consider.

—Guang-Zhong Yang

in minimally invasive surgery, including motion scaling, improved hand–eye coordination with aligned visual–motor axes, 3D vision and improved instrument control and dexterity using endo-wrists. These developments represent major milestones in the engineering development of surgical robots. Although their initial clinical uptake was slow, they opened a brand new chapter in modern surgery.

As the clinical use of surgical robots started to gain traction, it was clear that general-purpose surgical robots may not be practical for different clinical specialties as we know, for example, that the requirement for minimally invasive cardiothoracic operations can be very different from that of urological operations. To this end, the third-generation surgical robots started to emerge. They are dedicated surgical robots optimized for different clinical specialties, e.g. for spinal, endovascular, endobronchial and gastrointestinal procedures. The current, i.e. the fourth-generation, surgical robots can seamlessly integrate pre- and intra-operative imaging, real-time navigation and other advanced functions during surgery. They can operate through either multi-port or single-port settings.

The next generation, i.e. the fifth-generation surgical robot, is closely related to the clinical drive of personalized medicine and precision surgery, which calls for early diagnosis and treatment. With the continuing development of new diagnostic and imaging technologies, many diseases can be detected at an early stage such that minimally invasive techniques via endoluminal routes can be deployed. The sooner we intervene, the better the prognosis for the patient. This also has important medical and economic implications as less trauma, shorter hospital stay and quicker recovery would lead to a much-reduced healthcare burden, both for society and for the patients.

Early diagnosis and treatment will inevitably involve small but deep-seated lesions. If it is possible to reach these lesions through natural orifices, e.g. the airways, digestive or urinary tracts, it would mean no surface incisions, further reducing the surgical-access trauma. This is beneficial for elderly patients such as octogenarians or those with comorbidities. It would also mean that diagnostic procedures and intervention can be combined into one setting, rather than repeated hospital visits, further reducing the pressure on our healthcare systems, allowing many otherwise difficult procedures to be carried out in districted hospitals.

My personal research now in robotic surgery mainly focuses on the development of miniaturized fibrebots integrated with imaging, sensing and actuation that can be used for a range of endoluminal procedures. Another area that we are also working on is related to neurological intervention, including the development robots for deep-brain stimulation and brain–computer interfacing.

## TRI-CO ROBOTS

***NSR:*** What is your perspective on Tri-Co Robots (Coexisting–Cooperative–Cognitive Robots)? As one of the current leading issues and today's main topic, what are your suggestions and anticipations toward the development of Tri-Co Robots?

***Yang:*** Human–robot symbiosis has always been an important topic in robotics. As you know, it is a difficult task for robots to completely replace people in many fields, especially in complex environments. Hence, how to optimize the collective capability or behaviour is what Tri-Co Robots should consider.

From the perspective of its research and development, major challenges in Tri-Co Robots include the following.

First, there are challenges in design. These are about how to make the robot rigid when needed yet flexible enough for interacting with the environment. This means its actions are both adaptable and controllable. Specific research issues include mechanism design, new materials and fabrication schemes, analysis and modelling of control and mechanical behaviours in a coupled rigid–flexible setting. These are some of the example research issues that need to be addressed to ensure safe, versatile and intelligent mutual interactions in different application scenarios.

Second, there are challenges in interaction. These concern the interaction between human, robot and the environment, especially interactions related to human perceptions. About 15 years ago, I proposed the conception of Perceptual Docking, which is closely related to Tri-Co Robots. It represents a fundamental paradigm shift of perceptual learning and knowledge acquisition for robotic systems in that operator-specific motor and perceptual/cognitive behaviour is assimilated *in situ* through implicit interactions rather than traditional pre-programming. In the Tri-Co Robots world, humans and robots are expected to work together in dealing with complex tasks, collaboratively, adaptively and intelligently. Everyone has his/her specific set of approaches, experiences and behavioural patterns when facing a specific situation. But when it comes to a robot handling a less familiar task or working in a new environment, the importance of mutual understanding between human and the robot should be emphasized. Existing techniques such as deep learning often turns robot learning into a black-box system, leading to unilateral or incomplete understanding in the robot's behaviour. While it is sometimes difficult for us to understand robots, it seems even more difficult to teach robots to understand us human beings. When I was at Imperial College London, we developed a gaze-contingent surgical robot that can adaptively learn from a surgeon's behaviour and collaborate, not via pre-programming but real-time *in situ* learning, with the operator. This mutual understanding is an important part of developing Tri-Co Robots.

Third, there are challenges in deployment. These are related to the application of robots in daily life. As mentioned earlier, issues related to potential regulatory, ethical and legal barriers must be addressed as we see increasing use of robots in our society. This is even more so for Tri-Co Robots when deployed in close collaboration with humans.

Of course, there are many other basic science and engineering challenges related to the field. Our journal *Science Robotics* in fact covers many aspects of these challenges.

***NSR:*** From the perspective of scientific research and applications, what are some notable focuses in the sphere of robot intellectualization currently?

***Yang:*** Generalized intelligence for robots is difficult. When enough data are available for training, it is possible to overcome many of the challenges in machine learning. In terms of research opportunities, explainable AI and generalized learning, particularly unsupervised paradigms, would be worth exploring. Many automatic driving systems have now reached to an autonomy level of L3 and L4 (with six in total, L0–L5). From a scientific-research standpoint, there are also other challenges related to autonomy under uncertain and changing environments, or when there is a lack of concrete samples for learning. How to incorporate high-level knowledge and priors to derive a truly intelligent yet generalizable, explainable and adaptable would be an interesting research direction to pursue.

***NSR:*** In addition to intelligence, perception and driving are also very important directions for robot development. What do you think is worthy of attention in the direction of perception and drive?

***Yang:*** For perception, I think an important point now is combination with brain–computer interfacing (BCI), which has been an issue of great interest to us. Another problem in perception is how to effectively combine human cognition with robot perception. I mentioned that through a detailed analysis of eye movements, it is possible to gauge intention from the user implicitly, allowing more seamless human–robot interaction. Other perceptual feedback routes such as haptic and tactile are also valuable cues to incorporate. These can also be combined with measurement of cortical activities, through non-invasive BCI techniques such as EEG and fNIRS.

New sensing and imaging techniques would play an important role in the future development of robotics, particularly for them to co-exist within our daily living environment. The development of soft-robotics and e-skin for robotics is an example of this. In addition, for wearable robotics, such as exo-skeleton robots, we will see their increased use in heavy lifting, rehabilitation and assistive scenarios. The development of soft-exoskeletons by leveraging the new development in materials is an area that we are particularly interested in. At our institute, we are investigating the use of smart fibres and new actuation

schemes for this purpose. In *Science Robotics*, we have organized several editorials and special sections on the development of new materials for next-generation robots.

## ROBOTS FOR THE FUTURE

***NSR:*** We all have a lot of good expectations of robots—what do you think are the differences between reality and imagination? What are the challenges from a technical point of view?

***Yang:*** The public's expectations of robots are closely related to our new scientific and engineering breakthroughs, and our ability to translate these advances into tangible applications.

For example, the public's initial perception of robots probably originates from science-fiction movies, in which robots are often omnipotent, in terms of perception, intelligence and operation, especially for humanoid robots. In real life, some exciting developments in humanoid robots have emerged. We have seen examples by Boston Dynamics and other humanoid robots from research institutions and companies in China, Japan and Europe. Compared with humans, however, they are still far from those portrayed in movies in terms of general capabilities, agility and intelligence.

Robots certainly have many advantages over humans, particularly in terms of power, accuracy and repeatability. However, it is important to note, particularly for the general public, that certain tasks that we take for granted may in fact be very difficult for robots. For example, ironing clothes is generally considered to be a very simple task and a child can fold clothes neatly without much training. However, handling soft, flexible and deformable objects is challenging for robots. In May 2021, *Science Robotics* organized a special issue on robot grasping, within which we highlighted the challenges of modelling, learning, perception and control methods for manipulating deformable objects. I think we roboticists also have the responsibility in public outreach to effectively communicate to the general public and policy makers about the real challenges and factual advances of robotics, rather than riding on the hype and fuelling the public with unrealistic expectations.

Another example is computer vision. In the early years of AI, everyone thought that computer vision should not be a big deal for robotics as a 3-year-old could navigate freely in new environments and manipulate complex objects seen or unseen before. We soon realize how difficult it is for a machine to accurately recover motion and depth, and understand complex scenes. Computer vision is now a highly specialized yet multidisciplinary area. The realization of autonomous driving is largely built on the advances of computer vision over the past few decades.

We need to realize that robotics is a multidisciplinary but relatively young field. There remain many unresolved technical challenges and it is important to be realistic about our current technical capabilities and take tangible steps in advancing the field as a whole.

I would encourage our roboticists to think originally rather than just following the waves or pursuing what is trendy, with serious consideration of societal responsibilities.

—Guang-Zhong Yang

***NSR:*** With the development of robotic techniques, what are your thoughts concerning the relationship between humans and robots in the future?

***Yang:*** Bill Gates once mentioned ‘the disappearing computers’ to predict how digital computers would become part of our lives. Of course, what he meant by ‘disappearing’ is really to suggest that computers will one day become ‘pervasive’, ‘ubiquitous’ or ‘omnipresent’. I think the same would be true of robots. In my inaugural editorial for *Science Robotics*, I mentioned that the problems prompting society to develop robotic systems to replace or extend human presence are unlikely to abate; robots will increasingly be intertwined with us economically, technically and also socially.

***NSR:*** From your point of view, what are the features of the research and application of robots in China? What are your expectations for the future development of robots in China?

***Yang:*** The field of robotics, in terms of both fundamental research and practical applications, has witnessed tremendous growth in China in recent years. Compared to those in other countries, people in China are more receptive to robots, willing to embrace them for all walks of life and explore their new applications, often with great enthusiasm and passion. Robots are now being widely used in many aspects of manufacturing, logistics and healthcare. In recent years, the central government established a series of National Key R&D Programs to give support for robotics research and development, which is further leveraged by increasing investment from the private sector to high-tech start-ups. This is a positive sign for promoting robot techniques nationally. During the last Winter Olympic Games, for example, robots seemed to be everywhere, from the Olympic torch relay, broadcasting, logistics and security inspection, to automated disinfection and smart catering. If you are interested, there is a focus article in *Science Robotics* describing how robots were used in Beijing 2022.

As for my expectations or advice for the future development of robotics in China, I think it is worth considering the following. First, we should focus more on original research and fundamental theoretical and engineering development, and having the courage and determination to do so. Only through sustained effort in fundamental research can we transform the robotics landscape in China, being a leader rather than a follower.

Second, we need to leverage the unique strength of our nation's R&D infrastructure to tackle major scientific challenges on a scale that is difficult for other nations. The innovative use of robotics for the space programme is one example. The same is true for the use of robotics for healthcare. I am impressed by myriads of start-up companies in developing different new surgical robots across the country in recent years.

Third, when we develop new technologies, it is vital to ensure that there would be tangible socio-economic benefits. Take medical robotics as an example: how to lower the cost and make the technology benefit the population at large, rather than the privileged few, would be an important factor to consider.

Finally, I would encourage our roboticists to think originally rather than just following the waves or pursuing what is trendy, with serious consideration of societal responsibilities. For example, I have been advocating more attention to be paid to agricultural robotics in anticipation of the future development of precision farming and the potential lack of labour force in agriculture due to the aging population. Precision agriculture is not simply about robots picking apples or being used for wheat harvesting; rather, it considers how to reduce the use of chemical fertilizers, weed killers and pesticides, as well as how to ensure soil safety and better protect our water resources. Therefore, it would have a significant impact on our environment and natural resources, directly and indirectly. Linking technological development with national foresight is important to the future development of robotics in China.

